# Transient narrowing of a wide QRS tachycardia. What is the mechanism?

**DOI:** 10.1002/joa3.12334

**Published:** 2020-03-24

**Authors:** Francisco Ribes, Ángel Martínez‐Brótons, Ángel Ferrero‐De‐Loma‐Osorio, Lourdes Bondanza, Cristina Albiach, Víctor Marcos‐Garcés, Ricardo Ruiz‐Granell

**Affiliations:** ^1^ Arrhythmia Unit Department of Cardiology Hospital Clínico Universitario Valencia Valencia Spain; ^2^ Instituto de Investigación Sanitaria INCLIVA Valencia Spain

**Keywords:** accessory pathway, concealed conduction, orthodromic atrioventricular reentrant tachycardia, wide QRS tachycardia

## Abstract

Electrocardiogram showing a regular wide QRS tachycardia with left branch block (LBBB) like in morphology at 200 beats per minute (bpm). During electrophysiology study, it suddenly gets narrow and faster. What is the mechanism of the switch from a broad complex to a narrow complex tachycardia?

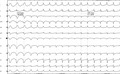

## 1 CASE PRESENTATION

**Figure 1:**
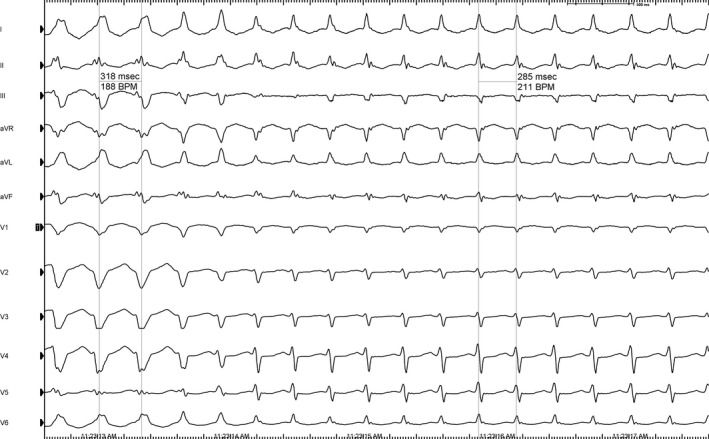
Twelve lead electrocardiogram (50 mm/s). Spontaneous changing in the QRS duration leads to an abridgement of the tachycardia cycle length.

A 56‐year‐old male was admitted to the Emergency Department for palpitations with no other background. The electrocardiogram (ECG) showed a regular wide QRS tachycardia with left bundle branch block (LBBB) like in morphology at 200 beats per minute (bpm). A rapid bolus of adenosine interrupted the tachycardia and sinus rhythm was restored. During the electrophysiology study, the tachycardia was spontaneously induced (Figure 1). What is the mechanism of the switch from a broad complex to a narrow complex tachycardia?

## 2 DISCUSSION

As shown in Figure 1, R‐R intervals during LBBB‐like beats are longer than R‐R intervals while narrow complexes. Such observation, rules out phase 3 and phase 4 block phenomenon. We hypothesize that a temporary shortening of the effective refractory period (ERP) of the LBBB could lead to a conduction over both branches, with a narrow complex.

**Figure 2:**
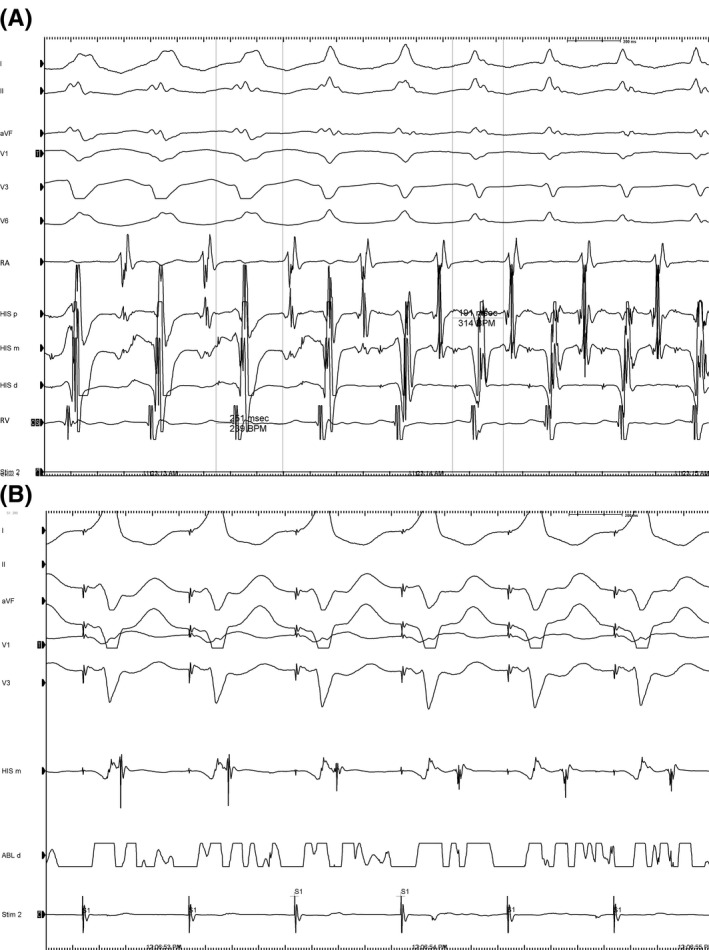
A, During wide QRS tachycardia (LBBB), HA interval (from the first H to the first atrium registered) measures 251 ms. Transient narrowing of the QRS predicts a subsequent shortening of the HA interval (191 ms). Note the change from a wide QRS to a narrow QRS indicated by arrows. RA, right atrium; His, His bundle recordings from proximal to distal; RV, right ventricle; LBBB, left bundle branch block. B, During ablation, ventricular and atrial electrograms merge together while ventricular pacing (red arrow). After the third beat, the atrial electrogram is completely unmasked after successful ablation (green arrow). Then, atrial activation occurs exclusively over de AV node. His m, His bundle recording; ABL d, ablation recording.

For the electrophysiology study, two tetrapolar diagnostic catheters were placed in the right ventricule apex and in the His bundle region. Despite many attempts to reach de coronary sinus, it was impossible to locate a diagnostic catheter inside it from a femoral approach, probably being an hypertrophic Thebesian valve the underlying cause. In Figure 2A, we can see the intracardiac tracing showing an AV 1:1 relation. The progressive narrowing of the QRS with subsequent shortening in the tachycardia cycle length which predicts changings in the next A‐A interval,^1^ proves the dependence of the His‐Purkinje system or ventricular myocardium in the tachycardia circuit.

Accordingly to these findings, the differential to this tachycardia would be a ventricular tachycardia with ventriculoatrial (VA) conduction or an atrioventricular reentrant tachycardia (AVRT), and hence, the diagnostic clue would be the His Bundle (HB) activation pattern. Thorough examination of the tracing shown in Figure 2A, reveals a HB activation preceding both the local ventricular electrogram and the QRS‐onset. Moreover, HV interval during tachycardia is almost identical to the one recorded in sinus rhythm (HV: 44 milliseconds).^2^ These observations are consistent with an orthodromic atrioventricular reentrant tachycardia because of a concealed accessory pathway.

As shown in Figure 2A, during AVRT activation wavefront from the His‐Purkinje system to the first atrium electrogram recorded while LBBB‐like beats is longer than the same interval while narrow QRS beats. Narrow QRS beats suggest simultaneous activation of both branches of the His whereas LBBB‐like beats are conducted down over the right bundle, with an enlargement in the tachycardia circuit. Functional bundle branch block (BBB) ipsilateral to an accessory AV pathway forces the reentrant circuit to follow a longer path and hence, more time is needed for the tachycardia wavefront to travel from the AV node down the HB and contralateral bundle branch, and then toward the myocardium to the ventricle ipsilateral to the BBB in order to reach the accessory pathway and then activate the first atrium.^3^ Such observation proves the presence and participation of a left‐sided accessory pathway in the tachycardia.

A transseptal puncture was performed. Mitral annulus mapping with the ablation catheter during tachycardia identified atrial insertion of the bypass tract in the left free wall. The tachycardia was interrupted spontaneously. Then, ablation was performed during ventricular pacing (Figure 2B). This tracing reveals almost simultaneous atrial and ventricular electrograms, meaning VA conduction over the accessory pathway and presumably over the AV node.^4^, ^5^ Radiofrequency application at this site successfully eliminated the bypass tract and the patient had no further recurrence.

This case highlights the importance of thorough study of the tachycardia tracing during differential diagnosis of a wide QRS tachycardia. It shows the importance of classical maneuvers in electrophysiology studies, which can lead us to the definitive diagnosis with only a few catheters, even in challenging tachycardias.

## CONFLICT OF INTERESTS

All authors had access to the data and a role in writing the manuscript.
